# Peroxisome proliferator-activated receptor-α activation facilitates contextual fear extinction and modulates intrinsic excitability of dentate gyrus neurons

**DOI:** 10.1038/s41398-023-02496-1

**Published:** 2023-06-15

**Authors:** Guo Xiang, Xia Liu, Jiangong Wang, Shunshun Lu, Meng Yu, Yuhan Zhang, Bin Sun, Bin Huang, Xin-Yun Lu, Xingang Li, Di Zhang

**Affiliations:** 1 Department of Neurosurgery, Qilu Hospital and Institute of Brain and Brain-Inspired Science, Shandong University, Jinan, 250012 China; 2 Jinan Microecological Biomedicine Shandong Laboratory, Jinan, 250012 China; 3 Institute of Metabolic and Neuropsychiatric Disorders, Binzhou Medical University Hospital, Binzhou, 256600 China; 4 National Glycoengineering Research Center, Shandong University, Jinan, 250012 China; 5 Department of Neuroscience & Regenerative Medicine, Medical College of Georgia at Augusta University, Augusta, GA USA

**Keywords:** Hippocampus, Psychiatric disorders

## Abstract

The dentate gyrus (DG) of the hippocampus encodes contextual information associated with fear, and cell activity in the DG is required for acquisition and extinction of contextual fear. However, the underlying molecular mechanisms are not fully understood. Here we show that mice deficient for peroxisome proliferator-activated receptor-α (PPARα) exhibited a slower rate of contextual fear extinction. Furthermore, selective deletion of PPARα in the DG attenuated, while activation of PPARα in the DG by local infusion of aspirin facilitated extinction of contextual fear. The intrinsic excitability of DG granule neurons was reduced by PPARα deficiency but increased by activation of PPARα with aspirin. Using RNA-Seq transcriptome we found that the transcription level of neuropeptide S receptor 1 (Npsr1) was tightly correlated with PPARα activation. Our results provide evidence that PPARα plays an important role in regulating DG neuronal excitability and contextual fear extinction.

## Introduction

Post-traumatic stress disorder (PTSD) is a devastating mental health condition characterized by intense and intrusive feelings of fear associated with experiencing or witnessing traumatic events. The persistent expression of fear memory and the impairment in fear extinction due to the incapability to modulate fear expression using contextual information could contribute to the development of PTSD symptoms [1–3]. The hippocampus is critical for processing contextual information linked with fear and extinction memories [4–8]. Studies in humans revealed that PTSD is associated with volumetric reduction of the hippocampus, particularly the dentate gyrus [9–12].

The dentate gyrus (DG), located at the entrance of the hippocampal formation, is essential for contextual fear processing [13, 14]. Acquisition and expression of contextual fear recruit unique DG granule neuron ensembles to form engram of memory and activation of such engram is sufficient to induce freezing responses [15, 16]. Intriguingly, distinct ensembles of DG granule neurons are activated during contextual fear extinction and inhibition of DG activity or silencing the extinction-recruited DG granule neuron ensembles significantly impairs contextual fear extinction [14, 17, 18], suggesting that neuronal activities in the DG are also required for memory extinction. Despite the important role of DG in regulating contextual fear, the regulatory mechanisms underlying such functions remain poorly understood.

Peroxisome proliferator-activated receptor α (PPARα) is a ligand-activated transcription factor belonging to the family of ligand-regulated nuclear receptors [19, 20]. Similar to other PPARs in the family, PPARα forms heterodimers with retinoid X receptor to bind to the specific promotor region referred to as PPAR response elements (PPREs) to regulate the transcription of target genes [21–23]. PPARα binds to a variety of ligands including arachidonic acid metabolites and synthetic fibrate drugs [24–26]. Interestingly, aspirin has recently been identified as a potent ligand binding to PPARα at the Tyr314 residue of its ligand-binding domain (LBD) [27]. Although abundant evidence exists that PPARα is involved in regulating various biological functions including lipid metabolism [28], inflammation [29, 30], immunity [30, 31], and antioxidation [32], its modulation on higher brain functions is not fully characterized. In the hippocampus, PPARα is expressed in all subregions including the DG [33], yet the functional role of PPAR in DG remains unclear.

In this study, we set out to test the role of PPARα in regulating contextual fear extinction and modulating the function of DG. Using classic contextual fear conditioning and extinction paradigm [34], we examined the contextual fear acquisition, retrieval, and extinction of PPARα deficient mice and mice with intra-DG infusion of PPARα agonist aspirin. To confirm whether PPARα act on DG to regulate contextual fear extinction, we used CRISPR-Cas9 genome editing to delete PPARα specifically in the DG and tested the mice for contextual fear conditioning and extinction. To further investigate the mechanisms underlying PPARα regulation of contextual fear, we employed whole-cell patch-clamp recordings to test the intrinsic excitability of DG granule neurons under the conditions of PPARα deficiency and activation by aspirin. In the last, we explored the downstream molecules possibly mediating the function of PPARα on contextual fear extinction through RNA-Seq transcriptome analysis.

## Materials and methods

### Animals

Adult wild-type C57BL/6J mice were purchased from Beijing Vital River Laboratory Animal Technology (Beijing, China). PPARα^−/−^ mice (008154) were purchased from the JAX laboratory. PPARα^+/−^ mice were intercrossed to generate PPARα^−/−^ mice and wild-type littermates. Cre-dependent Cas9 mice (026175) were purchased from the JAX laboratory and maintained as homozygous. All experiments were carried out using male mice. Mice were group housed (5/cage) under a 12:12 h light–dark cycle (lights on at 7:00 a.m.) with ad libitum access to food and water. The sample size was determined based on similar studies reported previously. In all experiments, animals were randomly assigned to respective groups and data were collected and analyzed by different experimenters double blinded. All procedures were performed under the guidelines approved by the Institutional Animal Care and Use Committees of Qilu Hospital of Shandong University.

### Drugs

Aspirin (Sigma-Aldrich, Shanghai, China) was dissolved in artificial cerebrospinal fluid (Leagene Biotechnology, Beijing, China).

### Guide RNA design and AAV production

Guide RNA sequences against exon 4 were purchased from Santa Cruz Biotechnology (Santa Cruz, CA, USA) and gRNA sequences against exon 6 were designed using the CRISPRtool. Selected sgRNA sequences for virus production are: 5′-CAGCACGGACGAGTCCCCCGGC-3′ and 5′-CCGGGTCATACTCGCGGGAAAGA-3′. The validity of the gRNAs was tested in vitro by co-transfection of plasmid expressing a gRNA and Cas9 endonuclease into N2A cells and the effective genomic editing was confirmed with Illumina sequencing reads. AAV virus containing selected gRNAs were packaged by OBiO Technology (Shanghai, China).

### Stereotaxic surgery

Stereotaxic surgery was carried out as previously described [35, 36]. Briefly, mice were anesthetized with isoflurane (3% induction and 1% maintenance). For intra-DG microinjections, a bilateral guide cannula were implanted into the DG (coordinates: AP = −2.1 mm, ML = ±1.5 mm, and DV = −1.5 mm from Bregma). Mice were individually housed after surgery and allowed to recover for at least a week. A total volume of 1 μl of aspirin (2 µg/µl) or artificial cerebral fluid (aCSF) was infused bilaterally into DG at the speed of 0.125μl/min followed by an additional 5 min to avoid backflow from the injector withdrawn. For intra-DG microinjection of virus, AAV2/9-Cre-mCherry (OBiO) or AAV2/9-gRNA-EGFP with concentrations of 5–9.8 × 10^12^ viral particles per ml were injected bilaterally into the DG (coordinates: AP= −2.1 mm, ML = ±1.5 mm and DV = −2.4 mm from Bregma). A total volume of 1.0 µl of AAV vectors (0.5 µl/side) was infused. Behavioral experiments were conducted 21 days after AAV injection.

### Behavioral tests

#### Contextual fear conditioning and extinction

Fear conditioning and extinction were carried out in the fear conditioning chambers for mouse (Coulbourn Instruments, Whitehall, PA, USA) as described previously [35]. During the fear conditioning, mice were allowed to explore the conditioning chamber for 180 sec followed by four electric foot shocks (2 sec, 0.75 mA, 60-s ITI). An additional 60 sec was given following the last shock before removing the mice from the context. Fear extinction was performed 24 h after fear conditioning during when the mice were re-exposed to the training context without electric foot shock for 5 min. Context-dependent freezing during the 5 min was recorded and analyzed with the Freezeframe3 software (Coulbourn Instruments, Whitehall, PA, USA). The chamber was cleaned with 20% ethanol between each run of the animals.

#### Anxiety tests

Open field test -The open arena (40 × 40 × 40 cm, white acrylic box) was divided into nine equal squares. The central square was defined as the center zone. Mice were placed in the center zone and activity was recorded for 10 min. The total distance traveled and the time spent in the center zone in the first 5 min was analyzed using the Limelight software (Coulbourn Instruments). Elevated plus maze – The maze was purchased from RWD (Shenzhen, China). The two open arms sized 30 × 5 cm and two closed arms sized 30 × 5 × 15 cm formed a shape of a “plus” sign. The maze was placed 70 cm above the floor. Mice were initially placed in the center area (5 × 5 cm) facing the corner and were allowed to explore the maze for 5 min. Exploratory activity was recorded and analyzed using ANY-maze software (Stoelting Co., Wood Dale, IL, USA).

#### Hot-plate test

Mice were placed on the hot plate (55 °C) and the latency of the first hind paw lick was recorded. The mouse that responded was removed immediately from the hot plate. If the mouse did not respond within 90 s, the test was terminated.

#### Visual cliff test

A clear acrylic box and a piece of clear glass were used to make the mouse visual cliff. The checkerboard paper was used to cover the inner surface of the box. A piece of clear glass was glued to the bottom of the box with half of its width suspended 70 cm above the floor, serving as the visual cliff. Each mouse was placed at the midline of the glass, and the side into which all four paws of mouse stepped was recorded. Each mouse was subjected to 10 consecutive trials.

### Whole-cell patch-clamp recordings

Whole-cell patch-clamp recordings were performed as previously described and all solutions were prepared freshly before use according to Zhang [35]. Briefly, adult male mice were anesthetized with isoflurane and then perfused with ice-cold perfusion solution. The brains were quickly transferred to an ice-cold cutting solution and the coronal slices (300 μm) containing the DG were prepared. The slices were incubated in an oxygenated (95% O_2_/5% CO_2_) extracellular solution at 25 °C for at least 1 h for recovery followed by constant perfusion with the extracellular bath solution. Granule neurons were visualized with oblique infrared illumination and clamped under the whole-cell mode using HEKA EPC10 feedback amplifier and patchmaster software (HEKA Instruments, Lambrecht/Pfalz, Germany). Recordings were made on cells which had a resting membrane potential of −70 mV and input resistance of 300 MΩ or larger. Aspirin was bath applied and multiple recordings were made at the time point of 0, 5, and 30 min. In order to maintain the integrity of cytoplasmic components during the 30 min recording, perforate patch clamp was used by adding 200 nm amphotericin B to the internal solution. To investigate the firing properties of neurons, current-clamp recordings were made from incremental positive current injections (10 pA) for a 1-s duration. Analysis of passive membrane properties of cells was made at the resting membrane potential. Input resistance was measured from a hyperpolarizing current injection of −40 pA. The AHP size was measured as the difference between the spike threshold and voltage minimum after the action potential peak. The spike amplitude, half-width and spike threshold were measured from the first spike during ramp stimulation.

### RNA-seq transcriptome analysis

The DG of PPARα^−/−^ mice (*n* = 4) and WT littermates (*n* = 4) were freshly collected and sequenced at Novagene Co. LTD (Beijing, China). The total RNA (1 μg) was extracted and cDNA libraries were created with NEBNext^®^ UltraTM RNA Library Prep Kit for Illumina^®^ (NEB, Beverly, MA, USA) according to the manufacturer’s recommendations. The samples were then subjected to Illumina sequencing at Novogene Bioinformatics Technology (Beijing, China). Genes from PPARα^−/−^ mice and WT littermates were considered to be differentially expressed at *p* < 0.05.

### Real-time quantitative PCR

Real-time quantitative PCR (qPCR) was used to further validate the RNA-seq data. Total RNA from freshly dissected DG was extracted with TRIzol (Thermo Fisher Scientific Inc., Waltham, MA, USA). The total RNA was reverse-transcribed into cDNA using ReverTra Ace qPCR RT kit (TOYOBO, Osaka, Japan). Quantitative PCR was performed with Fast SYBR Green Master Mix (Thermo Fisher Scientific) in the 384-well plates on the LC480II thermocycler (Roche, Germany). The expression of specific genes was normalized using the housekeeping gene GAPDH and the relative mRNA expression was presented with 2^−ΔΔCt^.

### Western blot

The dentate gyrus was dissected out on ice under dissecting microscope immediately after the behavioral test and homogenized with RIPA lysis buffer (Thermo Fisher Scientific) containing phosphatase inhibitors (Merck Chemicals (Shanghai), Shanghai, China). The Pierce Protein Assay Kit (Thermo Fisher Scientific) was used to determine the protein concentration and a total amount of 30 µg of protein was loaded to the SDS-PAGE gel. The proteins were then transferred to a PVDF membrane followed by incubation in the blocking buffer (5% dry milk and 0.1% Tween 20 in 1× Tris-buffered saline) and subsequently primary antibodies at 4 °C. Primary antibodies used in the study were as follows: anti-PPARα (1:1000, PA1-822A, Thermo Fisher Scientific), anti-Cas9 (1:1000, 19526, CST, Danvers, MA, USA), anti-GAPDH (1:1000, ab8245, Abcam, Cambridge, MA, USA). The membrane was washed and incubated in HRP conjugated secondary antibody the next day. Signals were visualized with the Chemi-Doc XRS+(Bio-Rad; Hercules, CA, USA) and quantified using Image J.

### Statistical analysis

Statistical significance was assessed by one-way ANOVA (with/without repeated measures), two-way ANOVA (with repeated measures), or two-tailed unpaired t-tests and paired *t*-tests where appropriate. Mice with freezing level less than 20% during the retrieval test (non-learner) and mice with incorrect cannula placement were removed from statistical analysis. Shapiro–Wilk test was employed to check the normality of continuous variable’s distribution and similar levels of variance were observed between groups. Bonferroni *post hoc* tests were conducted to determine the significant effects in ANOVAs. Results were considered significantly different when *p* < 0.05. All data were presented as means ± standard error of the mean (s.e.m.)

## Results

### PPARα regulates extinction of contextual fear

**Fig. 1:**
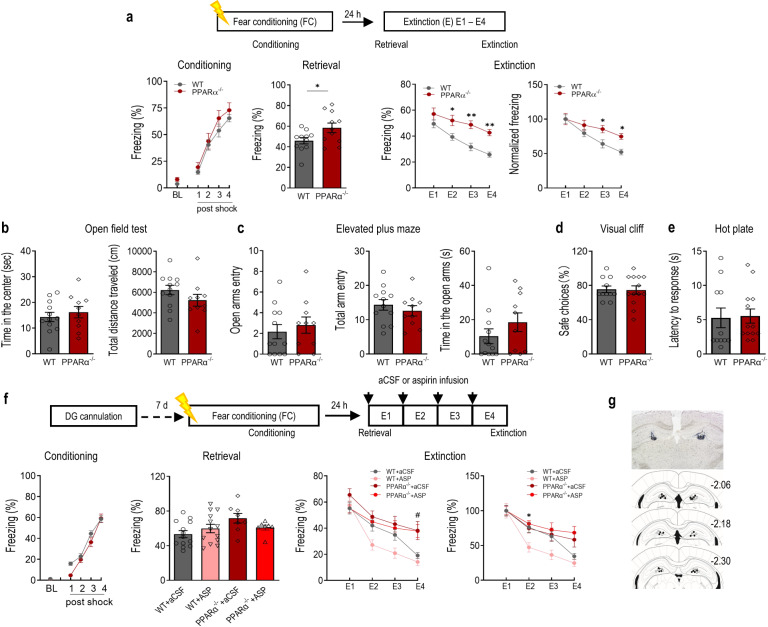
PPARα regulates contextual fear extinction. **a** PPARα deficiency impaired extinction of contextual fear. Upper, experimental time line. Lower, contextual fear acquisition (left, genotype: F (1,20) = 1.497, *p* = 0.2353; shock: F (80) = 90.08, *p* < 0.0001; genotype × shock: F (4, 80) = 0.3471, *p* = 0.8491), fear retrieval (middle left, t(20) = 2.262, *p* = 0.0350), fear extinction (middle right, genotype: F (1,20) = 13.36, *p* = 0.0016; day: F (3,60) = 29.19, *p* < 0.0001; genotype × day: F (3,60) = 2.219, *p* = 0.0951) and normalized fear extinction (F (1,20) = 4.207, *p* = 0.0536; day: F (3,60) = 29.95, *p* < 0.0001; genotype × day: F (3,60) = 3.416, *p* = 0.0229) in PPARα^−/−^ mice and WT littermates. PPARα^−/−^, *n* = 11; wild type (WT), *n* = 11. *p < 0.05, **p < 0.01 compared with WT littermates. **b** Open field test. Left, time spent in the center (t(20) = 0.6554, *p* = 0.5197); Right,. total distance traveled (t(20) = 1.366, *p* = 0.1870). **c** Elevated plus-maze test. Left, number of entries into the open arms (t(20) = 0.6109, *p* = 0.5482); Middle, total number of entries (t(20) = 0.7916, *p* = 0.4379); Right, time spent in the open arms (t(20) = 1.192, *p* = 0.2474). PPARα^−/−^, *n* = 10; wild type (WT), *n* = 12. **d** Visual cliff test. PPARα^−/−^ mice had normal visual depth perception compared with WT littermates (t(22) = 0.1308, *p* = 0.8971). **e** Hot-plate test. PPARα^−/−^ mice displayed similar paw withdrawal latency in reaction to heat stimulus compared with WT littermates (t(22) = 0.1534, *p* = 0.8795). PPARα^−/−^, *n* = 13; wild type (WT), *n* = 11. **f** Activation of PPARα by intra-DG infusion of aspirin facilitated contextual fear extinction. Upper, experimental time line. Lower, fear conditioning (left, genotype: F (1,39) = 2.646, *p* = 0.1119; shock: F (4,156) = 220.0, *p* < 0.0001; genotype × shock: F (4,156) = 2.647, *p* = 0.0356), fear retrieval (middle left, (F (3,37) = 2.476, *p* = 0.0765), fear extinction (middle right, treatment: F (3, 36) = 6.626, *p* = 0.0011; day: F (3, 108) = 45.40, *p* < 0.0001; treatment × day: F (9, 108) = 2.236, *p* = 0.0249). and normalized fear extinction (Right, treatment: F (3, 36) = 5.053, *p* = 0.0050; day: F (3, 108) = 45.16, *p* < 0.0001; treatment × day: F (9, 108) = 2.425, *p* = 0.0150). WT + aCSF, *n* = 13; WT + ASP, *n* = 13; PPARα^−/−^ + aCSF, *n* = 8; PPARα^−/−^ + ASP, *n* = 7. **g** Microinjection sites in the DG. Open circle: WT + aCSF; close circle: WT + ASP; open square: PPARα^−/−^ + aCSF; closed square: PPARα^−/−^ + ASP. ^*#*^*p* < 0.05 compared between WT + ASP and PPARα^−/−^ + ASP; *p < 0.05, compared between WT+aCSF and WT + ASP. Data are presented as mean ± s.e.m.

To investigate whether PPARα modulates contextual fear memory, PPARα^−/−^ mice and WT littermates were subjected to contextual fear conditioning (4-foot shocks; 0.75 mA, 2 s duration, 60 s inter-shock interval). Subsequently, all mice were trained for fear extinction consisting of a 5 min re-exposure to the conditioned context in the absence of foot shock daily for 4 days (E1–E4). During fear conditioning, PPARα^−/−^ mice showed a similar level of post-shock freezing in comparison with WT littermates indicating the formation of the association of the context-unconditioned stimulus association in both groups of mice. The retrieval of contextual fear was accessed with the freezing responses during the first 3-min of extinction training session E1. PPARα^−/−^ mice displayed enhanced fear retrieval compared with WT littermates. The elevated level of freezing was maintained throughout the subsequent days of extinction training in PPARα^−/−^ mice (E2–E4). Because PPARα deficiency resulted in significantly higher freezing responses during extinction training session E1, levels of freezing were normalized to that of E1 to compare the rate of extinction between the two genotypes. PPARα^−/−^ displayed a slower rate of extinction throughout the 4-day extinction training, indicating the significantly impaired contextual fear extinction. *Post hoc* analysis showed significantly higher levels of freezing in PPARα^−/−^ mice during the last two days of extinction training in comparison with WT littermates (Fig. 1a). Together, these results indicate that PPARα is crucial for both contextual fear memory retrieval and extinction. The innate anxiety level was accessed to exclude the possibility of anxiety-induced freezing response. In the open field test, neither the time spent in the center nor the total distance traveled was different between PPARα^−/−^ mice and WT littermates (Fig. 1b). The results of the elevated plus maze (EPM) showed no effect of genotype in the number of open arm entries, number of total arm entries, and time spent in the open arm (Fig. 1c). PPARα^−/−^ mice also displayed comparable visual perception (Fig. 1d) and normal nociceptive responses (Fig. 1e) when compared with WT littermates.

Given the above findings in PPARα^−/−^ mice, we then tested the effects of PPARα activation on contextual fear extinction. Aspirin serves as a strong ligand and activates PPARα by binding to its ligand-binding domain at the Tyr314 residue [27]. Literatures have already demonstrated that some of aspirin’s regulatory effects on central nervous system were mediated by PPARα [37]. We therefore tested whether activation of PPARα by aspirin could facilitate contextual fear extinction. Wild-type mice and PPARα^−/−^ mice were bilaterally cannulated in the dentate gyrus (DG) of the hippocampus and subjected to contextual fear conditioning. The DG was chosen based on its critical role in modulating contextual fear. Mice were given intra-DG infusion of either aspirin (2 μg/side) or artificial cerebrospinal fluid (aCSF) 30 min before extinction training for four consecutive days. Retrieval of the contextual memory was not affected by intra-DG infusion of aspirin in either WT or PPARα^−/−^ mice. The contextual fear extinction was significantly different throughout the 4-day extinction training across all groups. *Post hoc* analysis revealed a significant difference between WT + ASP and PPAR^−/−^ + ASP group on the fourth day of extinction training, suggesting that the deficits of PPARα^−/−^ mice in contextual fear extinction remained with intra-DG infusion of aspirin. When freezing responses were normalized to that of E1 for each treatment group, intra-DG infusion of aspirin significantly facilitated the rate of contextual fear extinction on the second day of extinction training in WT mice. However, the facilitating effects of intra-DG infusion of aspirin were not observed in PPARα^−/−^ mice (Fig. 1f, g). These results suggest that intra-DG fusion of aspirin promotes extinction of contextual fear memory while such effects are largely eliminated by PPARα deficiency. Taken together, these results revealed the crucial role of PPARα in regulating contextual fear extinction.

### PPARα in the dentate gyrus is required for extinction of contextual fear

**Fig. 2:**
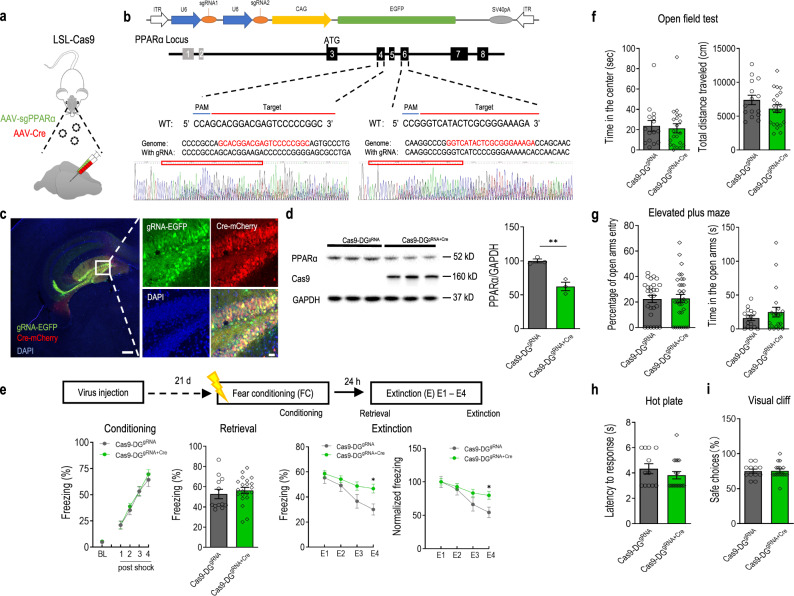
Deletion of PPARα in the dentate gyrus (DG) impairs contextual fear extinction. **a** Experimental procedure for DG-specific knockdown of PPARα using CRISPR-Cas9 system. Cre-dependent Cas9 mice were microinjected with AAV2/9-Cre and/or AAV2/9-sgPPARα into the DG. **b** Schematic of AAV vector and sgRNA design targeting the mouse PPARα locus. The Illumina sequencing reads showed the successful genomic editing of mouse PPARα gene with designed sgRNA. **c** Representative immunofluorescence images of Cre-dependent Cas9 mice with injection of AAV2/9-sgPPARα and AAV2/9-Cre into the DG. Green: sgPPARα; Red: Cre recombinase. Bar: 25 μm. **d** Representative immunoblot of the DG from Cre-dependent Cas9 mice microinjected with AAV2/9-sgPPARα (Cas9-DG^gRNA^) alone or together with AAV2/9-Cre (Cas9-DG^gRNA+Cre^) showed clear Cre-dependent induction of Cas9 expression and partial deletion of PPARα in the DG (t(6) = 3.059, *p* = 0.0222). **p < 0.01 compared with Cas9-DG^gRNA^ group. **e** DG-specific deletion of PPARα impairs contextual fear extinction. Upper, experimental time line. Lower, contextual fear acquisition (left, genotype: F (1,31) = 0.2453, *p* = 0.6239; shock: F (4,124) = 122.2, *p* < 0.0001; genotype × shock: F (4,124) = 0.2811, *p* = 0.8897), fear retrieval (middle left, t(31) = 0.6753, *p* = 0.5045), fear extinction (middle right, genotype: F (1,31) = 4.326, *p* = 0.0459; day: F (3,93) = 21.46, *p* < 0.0001; genotype × day: F (3,93) = 2.968, *p* = 0.0359) and the rate of extinction (right, genotype: F (1,31) = 2.202, *p* = 0.1479; day: F (3,93) = 22.32, *p* < 0.0001; genotype × day: F (3,93) = 3.489, *p* = 0.0188) in Cas9-DG^gRNA+Cre^. Cas9-DG^gRNA+Cre^, *n* = 21; Cas9-DG^gRNA^, *n* = 12. **f** Open field test. Left, time spent in the center, t(34) = 0.3557, *p* = 0.7243; Right, total distance traveled, t(34) = 1.430, *p* = 0.1618. **g** Elevated plus-maze test. Left, percentage of open arm entry (t(34) = 0.4754, *p* = 0.6376); Right, time spent in the open arms (t(34) = 0.9897, *p* =0.3293); Cas9-DG^gRNA+Cre^, *n* = 21; Cas9-DG^gRNA^, *n* = 15. **h** Hot-plate test. Cas9-DG^gRNA+Cre^ mice displayed similar paw withdrawal latency in reaction to heat stimulus in comparison with Cas9-DG^gRNA^ mice (t(27) = 1.070, *p* = 0.2942). **i** Visual cliff test. Cas9-DG^gRNA+Cre^ mice displayed normal visual depth perception compared with Cas9-DG^gRNA^ mice (t(27) = 0.07253, *p* = 0.9427). Cas9-DG^gRNA+Cre^, *n* = 17; Cas9-DG^gRNA^, *n* = 12. *p < 0.05 compared with Cas9-DG^gRNA^ control mice. Data are presented as mean ± s.e.m.

Based on the above findings, we reasoned that PPARα in the DG of the hippocampus is required for normal contextual fear extinction. To test this, we set out to delete the PPARα gene specifically in the DG using the CRISPR-Cas9 system through AAV-mediated expression of Cre and gRNA in Cre-dependent Cas9 mice (Fig. 2a). Guide RNAs that bind within exon 4 and 6 of the PPARα gene were constructed and validated. Illumina sequencing reads showed effective genomic editing (Fig. 2b). Selected gRNAs were cloned and packaged into an AAV vector co-expressing an EGFP reporter (AAV-sgRNA-CAG-EGFP). Injections of both AAV Cre and AAV gRNAs (Cas9-DG^gRNA+Cre^) or AAV gRNA (Cas9-DG^gRNA^) were then performed to delete PPARα in DG of Cre-dependent Cas9 mice. Immunofluorescence imaging showed the expression of both Cre recombinase and gRNAs in the DG (Fig. 2c). The knockout efficiency of PPARα protein in the DG was confirmed using western blotting. Compared to the Cas9-DG^gRNA^, Cas9-DG^gRNA+Cre^ mice displayed a significant reduction (~45%) in PPARα protein expression in the DG four weeks post-transduction (Fig. 2d).

To determine whether PPARα in the DG plays a role in regulating learned fear, Cas9-DG^gRNA+Cre^ and Cas9-DG^gRNA^ mice were subjected to contextual fear conditioning and extinction procedure. During conditioning, all mice showed an increase in freezing across conditioning trials, which did not differ between the two groups. Fear memory retrieval was also not affected by intra-DG knocking-down of PPARα when tested 24 h after fear conditioning. However, the contextual fear extinction was significantly different between the two groups. Cas9-DG^gRNA+Cre^ mice displayed significantly higher levels of freezing response as well as a slower rate of extinction on the fourth day of extinction compared with Cas9-DG^gRNA^ mice (Fig. 2e). These results suggest that PPARα in the DG is required for the formation of contextual fear extinction memory but not the acquisition or expression of original fear memory. Subsequent open field and EPM tests accessing the levels of anxiety were indifferent between Cas9-DG^gRNA+Cre^ and Cas9-DG^gRNA^ mice (Fig. 2f, g). The general visual and pain perceptions were also normal in Cas9-DG^gRNA+Cre^ mice compared with Cas9-DG^gRNA^ mice (Fig. 2h, i).

### PPARα knockout reduces intrinsic excitability of granule neurons in the dentate gyrus

**Fig. 3:**
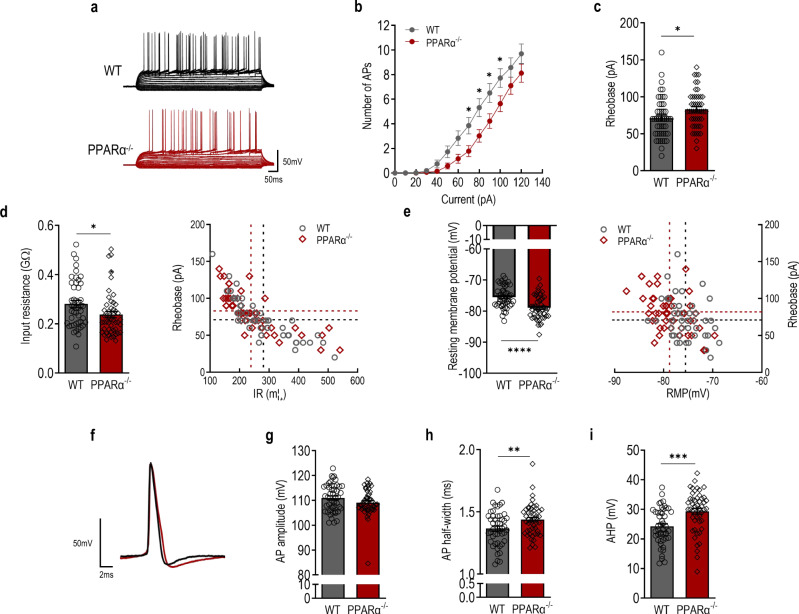
PPARα deficiency reduces intrinsic excitability of dentate gyrus (DG) granule neurons. **a** Action potential (AP) trains evoked by step-current injections in DG granule neurons of PPARα^−/−^ (red) and WT littermates (black). **b**. Number of APs (genotype: F (1,94) = 5.078, *p* = 0.0266; current: F (12,1128) = 169.9, *p* < 0.0001; genotype × current: F (12,1128) = 3.449, *p* < 0.0001). **c** Rheobase current (t(94) = 2.223, *p* = 0.0286). **d** Input resistance (IR). Left, input resistance (t(94) = 2.306, *p* = 0.0233). Right, IR vs rheobase of DG granule neorns. **e** Resting membrane potential. Left, resting membrane potential (t(94) = 4.627, *p* < 0.0001). Right, RMP vs rheobase of DG granule neurons. **f** Representative action potential waveform during ramp depolarization. **g** AP amplitude (t(94) = 1.653, *p* = 0.1016). **h** AP half-width (t(94) = 2.642, *p* = 0.0096). **i** Post-burst afterhyperpolarization (AHP)(t(94) = 3.822, *p* = 0.0002). PPARα^−/−^, *n* = 49 neurons from 5 mice; WT, *n* = 47 neurons from 5 mice. *p < 0.05, **p < 0.01, ***p < 0.001 and ****p < 0.0001 compared with WT littermate control. Data are presented as mean ± s.e.m.

DG granule neuron activity, particularly that of the memory engram cells, is crucial and delicately regulated during contextual fear extinction [14, 17, 38]. To explore the cellular mechanisms underlying PPARα regulation on contextual fear extinction, we tested whether PPARα modulates the intrinsic excitability of DG granule neurons. Whole-cell current-clamp recordings were performed on DG granule neurons of PPARα^−/−^ and WT littermates. DG granule neurons from PPARα^−/−^ mice displayed a significantly lower number of action potentials (APs) in response to the increment current injection (Fig. 3a, b). The decreased firing frequency was accompanied by increased rheobase current (Fig. 3c). To further examine the possible mechanisms modulating firing frequency in PPARα^−/−^ DG granule neurons, membrane properties and action potential waveforms were analyzed. The PPAR deficiency induced a decrease in input resistance and significantly lower resting membrane potential (Fig. 3d, e). Representative AP waveforms from DG granule neurons of PPARα^−/−^ and WT littermates are shown in Fig. 3f. The amplitude of APs from WT and PPARα^−/−^ mice were indifferent (Fig. 3g). The APs of PPARα^−/−^ mice were moderately but significantly wider than that of the WT littermates and they also displayed a larger afterhyperpolarization (AHP) (Fig. 3h, i). Taken together, these results suggest that PPARα plays a fundamental role in regulating the intrinsic excitability of DG granule neurons.

### PPARα mediates the effects of aspirin on granule neuron intrinsic excitability

**Fig. 4:**
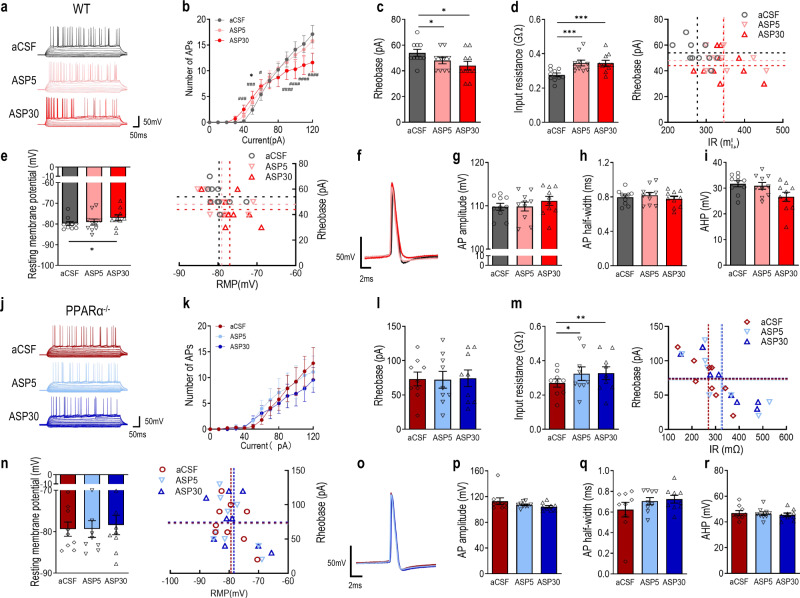
Aspirin modulates intrinsic excitability of dentate gyrus (DG) granule neurons through PPARα. **a** Action potential (AP) trains evoked by step-current injections in DG granule neurons of WT mice treated with aCSF (black) and 100 μM aspirin at 5 min (pink) or 30 min (red). **b** Number of Aps in aspirin treated DG granule neurons (treatment: F (2,180) = 3.023, *p* = 0.0511; current: F (9,90) = 24.41, *p* < 0.0001; treatment × current: F (18,180) = 4.349, *p* < 0.0001). *p < 0.05 compared with ASP5; ^##^*p* < 0.01 and ^####^*p* < 0.0001 compared with ASP30. **c** Rheobase current (F (2, 18) = 7.277, *p* = 0.0048). **d** Input resistance (IR). Left, input resistance (F (2, 18) = 14.38, *p* = 0.0002). Right, IR vs rheobase of DG granule neurons. **e** Resting membrane potential. Left, resting membrane potential (F (2, 18) = 4.081, *p* = 0.0346). Right, RMP vs rheobase of DG granule neurons. **f** Representative action potential waveform during ramp depolarization in DG granule neurons of WT mice treated with aCSF (black) and 100 μM aspirin at 5 min (pink) or 30 min (red). **g** AP amplitude (F (2, 18) = 1.005, *p* = 0.3857). **h** AP half-width (F (2, 18) = 4.206, *p* = 0.0317). **i** Post-burst afterhyperpolarization (AHP) (F (2, 18) = 6.014, *p* = 0.01). *n* = 10 neurons from 3 mice. *p < 0.05, **p < 0.01 and ***p < 0.001 compared with aCSF. **j** Action potential (AP) trains evoked by step-current injections in DG granule neurons of PPARα^−/−^ mice treated with aCSF (red) and 100 μM aspirin at 5 min (blue) or 30 min (navy). **k** Number of APs (treatment: F (2, 160) = 2.060, *p* = 0.1308; current: F (9, 80) = 5.849, *p* < 0.0001; treatment × current: F (18, 160) = 0.4932, *p* = 0.9582). **l** Rheobase current. F (2, 16) = 0.04061, *p* = 0.9603. **m** Input resistance. Left, input resistance (F (2, 16) = 5.205, *p* = 0.0182). Right, IR vs rheobase of DG granule neurons. **n** Resting membrane potential. Left, resting membrane potential (F (2, 16) = 0.4154, *p* = 0.6670). Right, RMP vs rheobase of DG granule neurons. **o** Representative action potential waveform during ramp depolarization in DG granule neurons treated with aCSF (red) and 100 μM aspirin at 5 min (blue) or 30 min (navy). **p** AP amplitude (F (2, 16) = 2.739, *p* = 0.0949). **q** AP half-width (F (2, 16) = 2.441, *p* = 0.1188). **r** post-burst afterhyperpolarization (AHP) (F (2, 16) = 2.283, *p* = 0.1342). *n* = 9 neurons from 3 mice. *p < 0.05 compared with aCSF. Data are presented as mean ± s.e.m.

Given the above findings that intra-DG infusion of aspirin promoted contextual fear extinction that could be facilitated by the neuronal activity of DG [38]. We further tested whether the activation of PAPRα by aspirin modulates the intrinsic excitability of DG granule neurons. Baseline firing of DG granule neurons from WT mouse brain slice was first recorded followed by bath application of 100 μM aspirin for 30 min. Recordings were then made at 5 min and 30 min of the incubation from the same patched neuron. The firing frequency of DG granule neurons was limited by two mechanisms: the repeated current injections induced an increase in the inter-spike intervals over time resulting in fewer action potentials or failures of action potential electrogenesis at higher current injection [39]. Bath application of aspirin for 30 min significantly increased the number of APs in response to depolarizing current injection between 0–80 pA, whereas at higher current injection, aspirin seemed to facilitate the failure of action potential electrogenesis (Fig. 4a, b). Decreased rheobase current (Fig. 4c), increased input resistance (Fig. 4d) and more depolarized resting membrane potential (Fig. 4e) were observed in DG granule neurons treated with aspirin for 30 min. Aspirin (30 min) treated DG granule neurons displayed unaltered AP amplitude and half-width as well as AHP (Fig. 4f–i). Notably, acute aspirin treatment for 5 min reduced the rheobase current and increased input resistance without affecting the number of APs induced with increment current injections and AP waveform. These results indicate that aspirin induces a two-phase modulation of intrinsic excitability of DG granule neurons. To further determine whether aspirin modulates DG granule neuron intrinsic excitability through PPARα, DG granule neurons from PPARα^−/−^ mouse brain slices were recorded under the same procedure. The effects of aspirin on the number of action potentials for either 5 min or 30 min during the 0 – 80 pA current injections were abolished in the brain slice of PPARα^−/−^ mice. (Fig. 4j, k). Rheobase (Fig. 4l) and resting membrane potential (Fig. 4n) were indifferent in groups with bath application of aspirin while input resistance was elevated in groups with bath application of aspirin. *Post hoc* analysis revealed an increase in input resistance in neurons treated with aspirin for 5 min and 30 min (Fig. 4m). Action potential waveforms including AP amplitude and half-width, as well as AHP, were not significantly altered by aspirin (Fig. 4o–q). Taken together, these results suggest that activation of PPARα mainly mediates the promoting effects of aspirin on the DG granule neuron excitability.

### Differentially expressed genes in the dentate gyrus upon PPARα activation

**Fig. 5:**
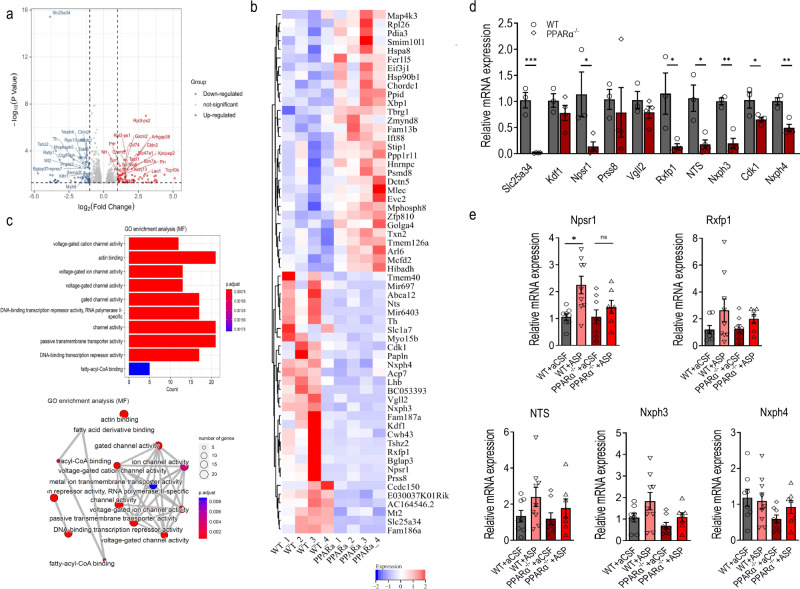
Differentially expressed genes upon PPARα activation. **a** Volcano plots (*p* value by fold changes (in log2 scale) of differential gene expression in the DG of PPARα^−/−^ mice (*n* = 4) and WT littermates (*n* = 4). **b** Heat map showing the top 30 most significantly upregulated or downregulated genes by PPARα deficiency. **c** GO enrichment analysis (MF) based on all differentially expressed genes. **d** Relative gene expression measured by quantitative real-time PCR of selected downregulated genes. Slc25a34: t(5) = 8.102 *p* = 0.0005; Kdf1: t(5) = 1.160 *p* = 0.2984; Npsr1: t(5) = 2.678 *p* = 0.0439; Prsss8: t(5) = 0.4249 *p* = 0.6886; Vgll2: t(5) = 1.181 *p* = 0.2908; Rxfp1: t(5) = 3.004 *p* = 0.0300; NTS: t(5) = 3.779 *p* = 0.0129; Nxph3: t(5) = 6.446 *p* = 0.0013; Cdk1: t(5) = 2.682 *p* = 0.0437; Nxph4: t(5) = 5.155 *p* = 0.0036. PPARα^−/−^, *n* = 4; WT, *n* = 3. *p < 0.05, **p < 0.01, ***p < 0.001 compared with WT. **e** Activation of PPARα by intra-DG aspirin infusion increased transcriptional expression of Npsr1. relative gene expression profiles of Npsr1, Rfxp1, NTS, Nxph3 and Nxph4 in WT and PPARα^−/−^ mice treated with intra-DG infusion of aCSF and aspirin. Npsr1: F (3, 28) = 5.169, *p* = 0.0057; Rxfp1: F (3, 28) = 1.693, *p* = 0.1911; NTS: F (3, 28) = 1.650, *p* = 0.2004; Nxph3: F (3, 28) = 3.276, *p* = 0.0356; Nxph4: F (3, 28) = 1.769, *p* = 0.1760; WT + aCSF, *n* = 8; WT + Aspirin, *n* = 9; PPARα^−/−^ + aCSF, *n* = 8, PPARα^−/−^ + Aspirin, *n* = 7. *p < 0.05 compared with WT + aCSF. Data are presented as mean ± s.e.m.

To explore the molecular mechanisms underlying the effects of PPARα on DG function and contextual fear extinction, we performed the RNA-seq transcriptome analysis on freshly dissected DG of PPARα^−/−^ mice and WT littermates. In the DG, the expression pattern of 913 genes were changed with PPARα deficiency with 389 genes upregulated and 524 genes downregulated (*p* value < 0.05)(Fig. 5a). Heat map of the top 30 upregulated or downregulated genes were illustrated in Fig. 5b. The analysis from molecular functions (MF) of Gene Ontology (GO) enrichment on all downregulated genes revealed that the differentially expressed genes (DEGs) were significantly enriched in voltage-gated cation channels, actin binding, passive transmembrane transport activity, DNA binding transcription repressor activity and fatty acyl-CoA binding (Fig. 5c). To further identify the possible downstream molecule that could be involved in PPARα regulation of contextual fear, we selected genes that were downregulated most dramatically with PPARα deficiency and the corresponding proteins were reported to be expressed in the DG for further confirmation using real-time quantitative PCR. Consistent with RNA-seq transcriptome analysis, we found that PPARα deficiency led to significantly lower levels of transcription of gene Slc25a34, Npsr1, Rxfp1, NTS, Nxph3 and Nxph4 in the DG (Fig. 5d). Among all the above-validated genes, Npsr1 and Rxfp1 belong to rhodopsin-like G-protein-coupled receptors (GPCR) subfamily A1 while Nxph3/4 belong to the neurexophilin family of neurexin ligands. These results indicate that PPARα in the DG plays an important role in regulating rhodopsin-like GPCR signaling and neurexin signaling. Given the above findings that activation of PPARα by aspirin increased neuronal excitability of DG granule neurons and facilitated contextual fear extinction, we then tested all the downregulated genes validated above in PPARα^−/−^ mice and WT littermates that were subjected to intra-DG infusion of aCSF or aspirin for 4 subsequent days during extinction. Real-time PCR results showed that among all genes probed, the transcription levels of Npsr1 were upregulated with intra-DG infusion of aspirin while such elevation of transcription were not significant in PPARα deficient mice (Fig. 5e). Taken together, these results suggest that PPARα could act on Npsr1 signaling pathways to regulate DG function and contextual fear extinction.

## Discussion

PPARα is well characterized for its regulation of lipid homeostasis in the peripheral tissues. However, its function and mechanism of action within the central nervous system are less well understood. Here, we provide direct evidence that PPARα acts on modulating the dentate gyrus (DG) granule neuron intrinsic excitability to regulate extinction of contextual fear. PPARα deficiency and deletion of PPARα specifically in the DG led to significant impairment in contextual fear extinction while activation of PPARα by intra-DG infusion of aspirin facilitated the rate of contextual fear extinction. Ex vivo patch-clamp recordings revealed that PPARα deficiency decreased while aspirin elevated DG granule neuron excitability. Further RNA-seq transcriptome analysis revealed that PPARα deficiency induced downregulation of neuropeptide S receptor 1 (Npsr1) in the DG while PPARα activation by aspirin upregulated their levels of expression. We believe these findings provide the first and direct evidence that PPARα plays important roles in the modulation of contextual fear processing and DG neuronal excitability.

Fear extinction, different from forgetting, is an active form of learning [40–42]. The newly formed extinction memory inhibits the retrieval and expression of previously acquired fear memories [41, 42]. PPARα has been shown to regulate cognitive functions including hippocampal-dependent learning and memory [43, 44]. PPARα null mice display impaired spatial learning and memory tested in a Barnes maze and PPARα mediated retinoid X receptor activation improves cognitive performance in an AD mouse model [33, 45]. Our study is consistent with these studies and complements them by showing that PPARα deficiency significantly impaired contextual fear extinction without affecting fear acquisition, suggesting that PPARα is required specifically for extinction learning in male mice. This is in line with what Locci and Pinna have reported that systematic administration of palmitoylethanolamine (PEA), the endogenous PPARα ligand, facilitates fear extinction in social isolated animals [46], confirming PPARα as an important regulatory molecule of fear extinction learning. In addition, our results further showed that deletion of PPARα in the DG led to a slower rate of extinction and activation of PPARα by intra-DG infusion of aspirin facilitated fear extinction. These results further target the DG of hippocampus as the key brain region through which PPARα exerts its role in regulating fear extinction. The function of DG is closely related to extinction of contextual fear. In the DG, the balance between the activities of fear encoding and fear extinction engram neurons determines the fear responses to the conditioning context [18]. Optogenetic inhibition of DG activity or silencing the extinction-recruited DG granule neuron ensembles significantly impairs contextual fear extinction, while enhancement of DG activity promotes contextual fear extinction, revealing the bidirectional control of contextual fear extinction by the DG [14, 18]. Furthermore, several lines of evidence also showed that modulating DG activity through genetic manipulation of proteins related to PPARα, such as adiponectin receptor 2 [47], and CKD5 [48] greatly affects extinction of contextual fear [35, 49], emphasizing the requirement of fine regulation of DG activity at the molecular level for normal fear extinction learning.

Interestingly, we also found that PPARα deficient mice exhibited higher freezing responses when tested for contextual fear retrieval, indicating the enhanced expression of original fear. To illustrate the effects of PPARα deficiency on contextual fear extinction per se, we have converted the levels of freezing to the percentage of that on E1 and PPARα deficient mice still displayed impaired fear extinction, indicating its possible role in regulating both stages of fear processes. Because the level of fear before extinction could influence the rate of subsequent extinction [50], we could not exclude the possibility that such high level of fear retrieval, rather than the protein itself, contributed to the impaired fear extinction exhibited in the PPARα deficient mice. However, the fact that mice with DG deletion of PPARα showed slower rate of extinction with normal level of fear retrieval, strongly suggests that PPARα in the DG regulates the extinction of contextual fear. PPARα is ubiquitously expressed in the brain [51], the enhanced fear retrieval is possibly attributed to function of PPARα in other brain regions of the fear circuitry. For example, Chikahisa et al have reported that PPARα null mice show enhanced fear learning in the passive avoidance test, due to the dysfunction of dopamine system in the amygdala [52]. PTSD is associated with enhanced fear and impaired fear extinction [53], both of which were observed in PPARα deficient mice, further illustrating the crucial role PPARα might play in the pathophysiology of PTSD. Notably, these results were obtained from male mice. Considering females have higher prevalence rates of PTSD [54, 55], the use of male mice only in our study limited the significance of the findings. Moreover, studies have shown that estrogen itself could inhibit the function of PPARα [28]. Therefore, future work is needed to clarify the gender differences in the modulatory effects of PPARα on PTSD-like behaviors.

The activity of DG is largely inhibited by strong, fast feedforward and feedback inhibition for efficient and precise transmission of contextual information [56]. Since it is difficult to induce synaptic plasticity in mature DG granule neurons [57–59], the plasticity of neuronal intrinsic excitability has thus become particularly important in modulating DG function. The intrinsic excitability of DG granule neurons is modulated by contextual fear and the levels of which directly influence the neuronal allocation to specific memory engram [60, 61]. We therefore focused to investigate the DG granule neuron excitability on brain slices. We found that DG granule neurons of PPARα deficient mice displayed significantly lower intrinsic excitability. The changes in neuronal excitability were reflected by increased rheobase current, decreased input resistance, more hyperpolarized resting membrane potential and larger AHP amplitude. Interestingly, when stimulating PPARα activity through bath application of aspirin, we revealed a biphasic regulation of intrinsic excitability of DG granule neurons, with an increased number of APs during lower current injections while a decreased number of APs during the higher current injections. The modulation of DG granule neuron excitability by aspirin was accompanied by the increased input resistance, more depolarized resting membrane potential and smaller AHP amplitude. PPARα deficiency blocked the upregulation of DG granule neuron excitability caused by aspirin as well as the resting membrane potential and AHP amplitude. The biphasic regulation of aspirin on DG granule neuron excitability indicates that multiple cellular mechanisms could be involved in mediating its action. Indeed, COX2, the classical target of aspirin is expressed in the adult DG granule neurons [62, 63]. Inhibition of COX2 reduces the postsynaptic membrane excitability through PGE2 [64], indicating that aspirin-induced decreased number of APs during the higher current injections could be mediated by its inhibition on COX2. These results together suggest that PPARα activity is required for maintaining DG granule neuron excitability through its modification of membrane properties.

To further investigate the molecular mechanisms that mediate effects of PPARα on DG function and contextual fear, we performed the RNA-seq transcriptome analysis in PPARα^−/−^ mice and WT littermates, as well as the real-time PCR from DG of mice treated with intra-DG infusion of aspirin. We found that the Neuropeptide S (Nps) signaling could be closely involved in mediating effects of PPARα on DG function and contextual fear extinction. The Neuropeptide S is a modulatory neuropeptide that composed of 20 amino acids. The synthesis of Nps is restricted to the peri-locus coeruleus area and the Kölliker–Fuse nucleus of the lateral parabrachial nucleus area in mice [65, 66], however, the Nps receptors are expressed in the several brain regions, among which some regions that lack Nps neuron projections [65, 67]. In the hippocampus, Npsr mRNA expression were only observed in the granule layers of the dentate gyrus [65]. We found that PPARα^−/−^ mice with lower mRNA expression of Npsr1 in the DG exhibited impaired extinction whereas when Npsr1 was upregulated with PPARα activation by aspirin, the mice showed facilitated fear extinction, suggesting its modulation of DG function and fear memory. Pharmacological studies in experimental animals revealed various functions of Nps in the central nervous system. Nps was found to induce arousal and wakefulness, reduce fear and anxiety, promote learning and memory consolidation, accelerate fear extinction, attenuate food intake and ameliorate cognitive impairments in WT animals or Alzheimer’s disease mouse models [67–74]. Interestingly, the results of the pharmacological studies have extended the function of Nps beyond its restricted projection areas, indicating the existence of Nps independent Npsr activation in regulating certain behaviors. The differential behavioral phenotype between the Nps deficient mice and Npsr deficient mice further supported this notion. The Nps precursor deficient mice displayed deficits in exploration and increased anxiety-related behavior [75] while Npsr1 knockout mice showed no significant impact on either locomotion or anxiety-related behavior [76–78]. In addition, the Npsr1 deficient mice showed deficits in various stages of contextual fear memory processing, including contextual fear generalization and context discrimination, further supporting the possible role of Npsr in the hippocampus independent of Nps [79, 80]. Studies have revealed that Nps modulates hippocampal synaptic plasticity through affecting the glutamatergic system [81]. In vitro pharmacological studies also showed that Nps acts as excitatory neurotransmitter and increases cellular excitability through Ca^2+^-dependent mechanisms [67, 82]. Together, these evidence suggest that Nps is capable of mediating the regulatory effects of PPARα on neuronal excitability as well as contextual fear extinction. Further studies are required to confirm its role in regulating DG function and mediating PPARα action.

In summary, our results provide direct evidence supporting a functional role of PPARα in modulating intrinsic excitability of DG granule neurons and extinction of contextual fear. We also identified a novel function of aspirin in regulating contextual fear extinction and DG function. In addition, our study further suggests the possibility of neuropeptide S system in regulating hippocampal function and contextual fear processing. Our current findings reported new cellular and molecular mechanisms underlying fear extinction and revealed the therapeutic potential of aspirin in facilitating extinction-based exposure treatments for PTSD and other stress-related disorders.

## Data Availability

The raw data of RNA-seq transcriptome datasets presented in this study was submitted online with the accession number listed as follows: NCBI SRA; PRJNA868499.
